# CircRNA Expression Profile during Yak Adipocyte Differentiation and Screen Potential circRNAs for Adipocyte Differentiation

**DOI:** 10.3390/genes11040414

**Published:** 2020-04-10

**Authors:** Yongfeng Zhang, Xian Guo, Jie Pei, Min Chu, Xuezhi Ding, Xiaoyun Wu, Chunnina Liang, Ping Yan

**Affiliations:** 1 State Key Laboratory of Grassland Agro-ecosystems, College of Pastoral Agriculture Science and Technology, Lanzhou University, Lanzhou 730020, China; zhangyongfeng_ying@163.com; 2 Key laboratory of yak Breeding Engineering Gansu Province, Lanzhou Institute of Husbandry and Pharmaceutical Sciences, Chinese Academy of Agricultural Sciences, Lanzhou 730050, China; guoxian@caas.cn (X.G.); peijie@caas.cn (J.P.); chumin@caas.cn (M.C.); dingxuezhi@caas.cn (X.D.); wuxiaoyun@caas.cn (X.W.); liangchunnina@caas.cn (C.L.)

**Keywords:** yak adipocyte, circRNA, adipocyte differentiation, RNA sequencing

## Abstract

The yak (*Bos grunniens*) is subjected to nutritional deficiency during the whole winter grazing season; deciphering the adipose metabolism and energy homeostasis under cold and nutrients stress conditions could be a novel way to understand the specific mechanism of energy metabolism. Circular RNAs (circRNAs) have elucidated that they play a key role in many biological events, but the regulatory function of adipose development remains mostly unknown. Therefore, the expression pattern of circRNAs were identified for the first time during yak adipocyte differentiation to gain insight into their potential functional involvement in bovine adipogenesis. We detected 7203 circRNA candidates, most of them contained at least two exons, and multiple circRNA isoforms could be generated from one parental gene. Analysis of differential expression circRNAs displayed that 136 circRNAs were differentially expressed at day 12 (Ad) after adipocyte differentiation, compared with the control at day 0 (Pread 0), while 7 circRNAs were detected on day 2. Sanger sequencing validated that six circRNAs had head-to-tail junction, and quantitative real-time PCR (qPCR) results revealed that the expression patterns of ten circRNAs were consistent with their expression levels from RNA-sequencing (RNA-seq) data. We further predicted the networks of circRNA-miRNA-gene based on miRNAs sponging by circRNAs, in which genes were participated in the adipocyte differentiation-related signaling pathways. After that, we constructed several adipocyte differentiation-related ceRNAs and revealed six circRNAs (novel_circ_0009127, novel_circ_0000628, novel_circ_0011513, novel_circ_0010775, novel_circ_0006981 and novel_circ_0001494) were related to adipogenesis. Furthermore, we analyzed the homology among yak, human and mouse circRNAs and found that 3536 yak circRNAs were homologous to human and mouse circRNAs. In conclusion, these findings provide a solid basis for the investigation of yak adipocyte differentiation-related circRNAs and serve as a great reference to study the energy metabolism of high-altitude animals.

## 1. Introduction

Yak is an iconic symbol of the Qinghai Tibetan Plateau [1]. Currently, there are more than 15 million yaks living on the Qinghai Tibetan Plateau, which represents about 90 percent of the world’s yak population. Yaks are essential for Tibetans and other nomadic pastoralists living in high-altitude surroundings and furnish the primary resources such as meat, milk, transportation, dung for fuel, and hides for tents [1]. The variations in the quality and quantity of natural herbage from season to season cause yak to become strong in summer, fatty in autumn, thin in winter, and tired in spring. The growth and survival of yak require the efficient storage of energy in adipose tissues. Yak store maximum energy in the adipose tissues during the summer season (June to September) when raised on lush pastures, this stored energy is utilized by yak to survive during the long cold season (October to May) [2]. Over the past several decades, abundant research has explored the molecular mechanisms of adipose tissue development and adipocyte differentiation. However, it remains a particularly fascinating topic in high-altitude species. Hence, exploration of the molecular mechanisms that modulate proliferation, differentiation, and metabolism of yak adipocytes may reveal a new clue to elucidate the development of adipose tissues in plateau animals.

CircRNA—non-coding RNA—has been recognized in recent years [3]. It is a class of RNAs that form covalently bounded closed-loop structures having neither 5’ to 3’ polarities nor polyadenylated tails [4] and exist abundantly in eukaryotes [5]. Previous studies verified that circRNAs are linked to cancer, atherosclerotic vascular disease, and neurological disorders in mammals; and circRNAs affect the neural development in mice, epithelial-mesenchymal transition in humans, and myogenesis in cattle [6,7,8]. Accordingly, evidence has shown that circRNAs can work as miRNA sponges to regulate gene expression, cell proliferation, differentiation, and apoptosis [9,10]. Consequently, we hypothesized that circRNAs, as a novel regulatory layer, may play a vital role during yak adipocyte differentiation.

For bovine adipogenic differentiation, non-coding RNAs of microRNA (miRNA) and long noncoding RNA (lncRNA) have been identified [11,12]. As another essential non-coding RNA, it remains to be elucidated whether circRNAs play a role in bovine adipocyte differentiation. No circRNAs investigated to date have been correlated with adipogenic differentiation of bovine adipocytes. In the current research, we used transcriptome sequencing to reveal the circRNA expression profile during yak adipocyte differentiation. Differentially expressed circRNAs were further screened out to learn more about their relation to yak adipocyte differentiation. Additionally, circRNA sequences were proven by Sanger sequencing at back-splicing site and by real-time reverse transcription PCR (qRT-PCR). The potential functions of circRNAs were analyzed by Kyoto Encyclopedia of Genes and Genomes (KEGG) in yak adipocyte differentiation. We aimed to identify circRNAs with a significant association with yak adipocyte differentiation for further research on adipose tissue development in yak. Our findings also provide a starting point to further explore the molecular mechanism of circRNAs in adipocytes of plateau animals.

## 2. Materials and Methods

### 2.1. Ethics Statement

The handling of animals during the experiment was carried out in strict accordance with the Animal Ethics Procedures and Guidelines of the People’s Republic of China, and all procedures were approved by the Animal Administration and Ethics Committee of Lanzhou Institute of Husbandry and Pharmaceutical Sciences of Chinese Academy of Agricultural Sciences (Permit No. SYXK-2014-0002).

### 2.2. Isolation of Pre-Adipocytes

Three healthy Datong yaks (3 days old) were offered by the Datong Yak Breeding Center (Datong County, Qinghai, China). Subcutaneous adipose tissue for cell isolation was harvested from three yaks at a local slaughterhouse and transported to the laboratory within sterile phosphate buffer saline (PBS) (HyClone, Thermo Fisher Scientific, Carlsbad, CA, USA) supplemented with 1% antibiotics (penicillin-streptomycin). Under a sterile environment, the potentially polluted epidermis, blood vessels and connective tissue were carefully removed. Subsequently, the remaining subcutaneous adipose tissue was washed with 1% antibiotic PBS several times. Then, samples were minced mechanically into approximately 1 mm^3^ section under sterile environment. The sections were digested by collagenase Type I for about 60–90 min at 37 °C with constant agitation. The digested tissues were filtered by 40 μm nylon mesh screen, and the filtrates were centrifuged at 1400 × *g* for 5 min. Subsequently, pellets were incubated with erythrocyte lysis buffer (0.154 M NH_4_Cl, 10 mM KHCO_3,_ 0.1 mM EDTA) at room temperature for 10 min and filtered with 200 μm mesh screen and washed twice with serum-free medium. The adipocytes were collected by centrifugation at 1400 × *g* for 5 min, cell counting was done with a haemocytometer, and then resuspended in pre-adipocyte growth media (DMEM-F12 supplemented with 10% fetal bovine serum). Thereafter, pre-adipocytes were maintained in 5% CO_2_ at 37 °C. After about 1 week, the cells were proliferated as pre-adipocyte naturally.

### 2.3. Adipogenic Differentiation and Oil Red O Staining Identification

In order to induce adipogenic differentiation, cells reaching to confluence in growth media were induced with adipogenic agents composed of 3-isobuty-methylxanthine (MIX) (Sigma, St Louis, MO,USA), dexamethasone (Sigma, St Louis, MO, USA), rosiglitazone (Sigma, St Louis, MO, USA) and insulin (Sigma, St Louis, MO, USA) for 2 days. Then, cells were fed with the culture medium, which was changed between 2–3 days intervals. Generally, cells were washed twice with PBS and fixed with 4% formalin for 1 h. Cells were stained with a saturated Oil Red O solution for 30 min at room temperature. Subsequently, the cells were washed three times with distilled water, and images were taken with light microscopy.

### 2.4. High-throughput RNA Sequencing of circRNAs Relevant to Yak Adipocyte Differentiation

TRIzol reagent was used to extract total RNA from in vitro cultured yak pre-adipocytes and differentiated adipocytes at days 0, 2 and 12 from three biological replicates for each condition. The quality and quantity of total RNA were analyzed by Qubit™ RNA Assay Kit in Qubit™ 2.0 Fluorometer (Life Technologies, Carlsbad, CA, USA) and NanoPhotometer^®^ spectrophotometer (Implen, Westlake Village, CA, USA), respectively. For one sample, approximately 5 μg RNA was used for RNA sample preparations. Ribozero™ rRNA Removal Kit (Epicentre, USA) was used to remove ribosomal RNA from the total RNA. To digest the linear RNA 3 U of RNase R was used. According to the manufacturer’s recommendations, NEBNext^®^ Ultra™ Directional RNA Library Prep Kit (NEB, Ipswich, MA, USA) was used to generate sequencing libraries for Illumina^®^. The libraries were sequenced on an Illumina Hiseq 4000 platform (Novogene, Beijing, China). For quality control, Q20, Q30 and GC contents of clean data were calculated. Subsequently, find_circ and CIRI2 [10,13] were performed to detect and identify circRNAs. The Circos software [14] was used to construct the circos figure. Back-splice algorithm picked out the junctions of unmapped reads. These were considered the reference sequence for next analysis and their expression was detected by TPM (transcripts per million clean tags) [15]. TPM was used to normalize the raw counts. Normalized expression level = (readCount × 1,000,000)/libsize (libsize is the sum of circRNA read count). The data have been uploaded to the Sequence Read Archive (SRA) database. The valid accession number is PRJNA550036.

### 2.5. Annotating Host Liner Transcript and Differentially Expressed circRNAs

Linear transcripts were annotated based on the location of the chromosome where the circRNA sequence was overlapped by HISAT2 (version 2.0.4) [16]. CircRNA distribution in the genome can be explored by comparing circRNA with genetic elements. Based on the negative binomial distribution test, the DESeq R package (1.10.1) [17] was used to detect the differential expression analysis of circRNAs. The same circRNAs in two yak adipocyte differentiation groups were used at day 0 (Pread 0) group as a control. The resulting *p*-values were adjusted using the Benjamini and Hochberg’s approach for controlling the false discovery rate. CircRNAs with an adjusted *p*-value < 0.05 were assigned as differentially expressed.

### 2.6. qRT-PCR Analysis for the Differential Expression of circRNAs

In order to verify the reliability of our analyzed data, ten differentially expressed circRNAs were selected. Total RNA was extracted from yak pre-adipocytes and differentiated adipocytes at days 0, 2, and 12 by using TRIzol reagent (Invitrogen, Carlsbad CA, USA) according to the manufacturer’s protocol. The expression levels of circRNAs were tested by qRT-PCR. The qRT-PCR was performed with a total volume of 20 μL containing 10 μL 2 x SYBR Premix ExTaq II (Takara, China), 0.5 μL each primer (10 μM) and 1 μL diluted cDNA. PCR conditions were as follows: 95 °C for 30 s; 40 cycles of 94 °C for 15 s, primer specific Tm for 30 s, and 72 °C for 30 s. The 2^−ΔΔCt^ method was used to calculate the relative expression profiles of circRNAs [18] with *β-actin* as a reference gene. CircRNAs amplified primers are listed in Supplementary Table S1.

### 2.7. Analysis of Enrichment and microRNA Target Site

GO seq R package was used for the Gene Ontology (GO, http://www.geneontology.org/) enrichment analysis for the host genes that showed differential expression of circRNAs [19]. If *p*-value ≤ 0.05, GO terms were regarded as significant enrichment. Kyoto Encyclopedia of Genes and Genomes (http://www.genome.jp/kegg/) database is a main resource for understanding high-level functions and utilities of biological systems. KOBAS software [20] was used to examine the statistical enrichment of genes or circRNA host genes that showed differential expression in KEGG pathways. MiRanda (http://www.microrna.org/) was used to identify the microRNA target site that located in exons of circRNA loci.

### 2.8. Co-expression Network Analysis of circRNA-miRNA-Gene during Yak Adipocyte Differentiation

The network of circRNA-miRNA-gene was established according to miRNA target site in exons of circRNA loci analysis. The putative correlations between circRNA, miRNA, and gene were ranked by miRnada and based on the hypergeometric distribution’s *p*-value. The networks were constructed by Cytoscape software (wersion 3.5.1, BiNGO plug-in) [21] in which circle nodes represented miRNAs and rectangle nodes represented genes and triangle nodes represented circRNAs.

### 2.9. Homology Analysis of circRNAs among Yak, Human and Mouse

The analysis of homology among yak, human and mouse was performed by BLAST (*e*-value < 1.0 × e^−5^) [22]. The circRNAs data of human and mouse were downloaded from circBase (http://www.circbase.org).

## 3. Results

### 3.1. High-throughput RNA Sequencing of Yak Pre-adipocytes and Adipocytes

**Figure 1:**
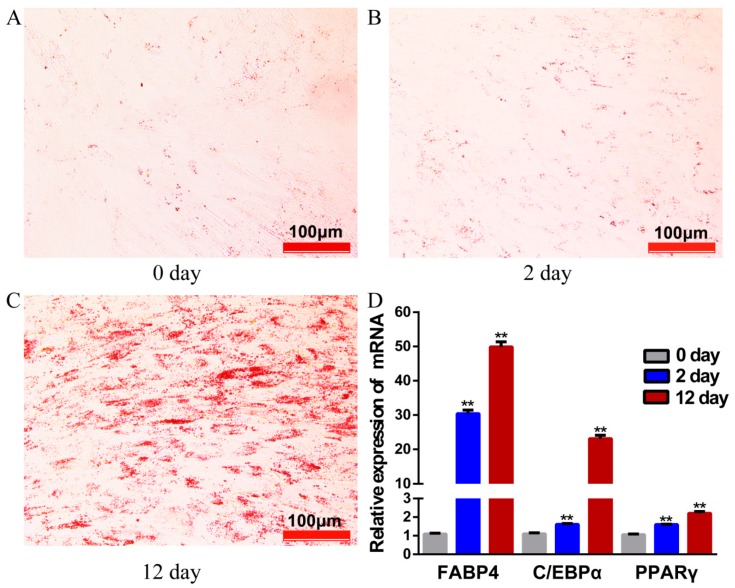
The yak preadipocytes and adipocytes for high-throughput sequencing. (**A**–**C**) Preadipocytes stained with Oil Red O on days 0, 2 and 12 of their differentiation. (**D**) The relative expression of genes PPARγ, C/EBPα and FABP4 detected by qRT- PCR. Values are means ± SEM(Standard Error of Mean) (*n* = 3); ** *p* < 0.01.

After 12 days of induction with adipogenic agents, lipid droplets visibility in adipocytes increased significantly, as stained with Oil Red O (Figure 1A–C). Adipocyte-specific marker genes PPARγ, C/EBPα, and FABP4, showed markedly higher expression on day 12 as compared to earlier stages (day 0, day 2) (Figure 1D), indicating that our model system met the functional criteria for yak adipocyte differentiation.

**Figure 2:**
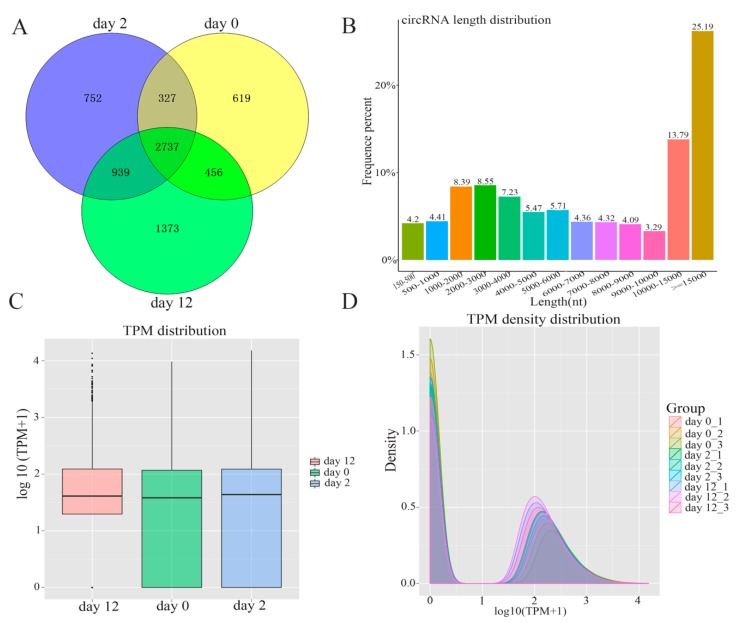
The expression pattern of circRNAs detected by high-throughput RNA sequencing during yak adipocyte differentiation. (**A**) Venn analysis of circRNAs detected at each time point. (**B**) The full-length distribution of circRNAs. (**C**) Box plots of transcripts per million clean tags (TPM) value for circRNAs in three groups. (**D**) The density distribution of circRNAs in pre-adipocytes and adipocytes.

In order to comprehensively identify the potential function of circRNAs during adipogenesis, high-throughput sequencing was performed to investigate the profiles of circRNAs by following a previously described pipeline [10]. The total numbers of reads obtained from nine samples are summarized in Supplementary Table S2. In total, 7203 circRNAs were identified during yak adipocyte differentiation at days 0, 2, and 12, of which 4139 circRNAs were detected at day 0, 4755 at day 2, 5505 at day 12, and 2737 at all three-time points (Figure 2A). The full length of adipocyte differentiation-related circRNAs ranged from 150 nt to over 15,000 nt. Among 7203 circRNAs, about 65.2% had a length of less than 15,000 nt, 8.61% had a length of less than 1000 nt, 29.64 % were between 1000 nt and 5000 nt in length, and 21.77% were between 5000 nt and 10,000 nt in length (Figure 2B). The expression abundance of circRNAs was measured based on TPM [15] during adipocyte differentiation, and the results indicated no abnormal expression in all the three groups (Figure 2C). This was highly consistent with the nine tested samples, as shown in Figure 2D.

### 3.2. Annotation of Host Linear Transcripts during Yak Adipocyte Differentiation

**Figure 3:**
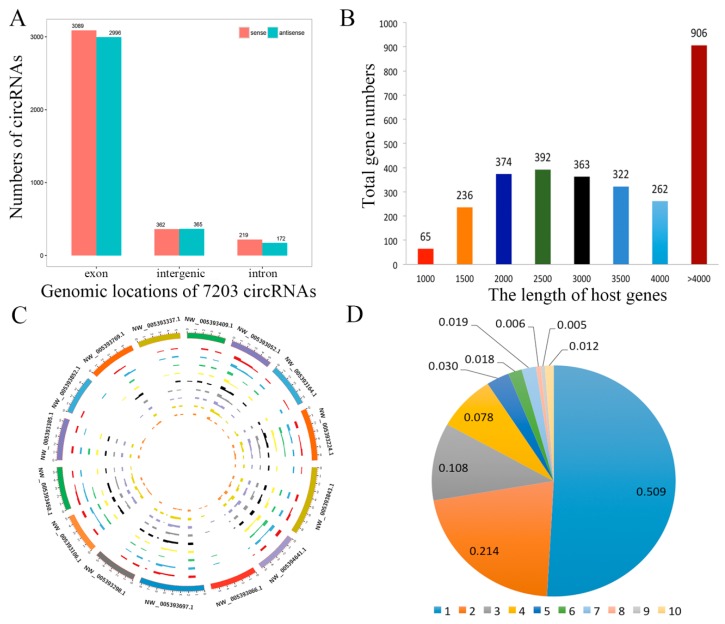
Profiling of circRNAs during yak adipocyte differentiation. (**A**) Distribution of circRNAs in the yak genome. (**B**) The length of host genes of circRNAs. (**C**) Circos plot showing the distribution of circRNAs in different chromosomes. (**D**) Numbers of circRNAs produced by the same gene.

CircRNAs biogenesis occurs through back splicing and then the canonical splicing of linear RNAs, and they are commonly formed by the alternative splicing of pre-mRNA. We annotated linear transcripts of circRNAs from the correlative genes which revealed a comprehensive landscape of the relationship between circRNAs biogenesis and their parental linear transcripts. Furthermore, we investigated the distribution of circRNAs in the genome, based on the overlapping sequences of circRNAs on chromosomes (Supplementary Table S3). The patterns of circRNAs distribution were characterized in the genome (Figure 3A). The mean transcript length of protein-coding genes is shown in Figure 3B. It revealed that the genomic loci of circRNAs were widely distributed across chromosomes (Figure 3C). In addition, we found that one parental gene could generate multiple circRNA isoforms, in this study, 7203 circRNAs were formed from only 2909 host genes (Figure 3D).

### 3.3. Expression Analysis of circRNAs during Yak Adipocyte Differentiation

**Figure 4:**
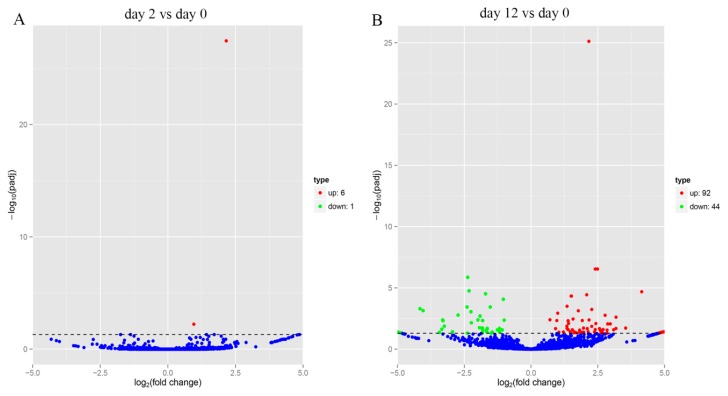
Differential expression analysis of circRNAs. (**A**) Volcano map of differentially expressed circRNAs between yak adipocyte differentiation at day 0 and day 2. (**B**) Volcano map of differentially expressed circRNAs between yak adipocyte differentiation at day 0 and day 12.

Comparison of the expression of circRNAs between two stages (day 0 and 12) indicated that 136 circRNAs were differentially expressed at 12 days after adipocyte differentiation, of which 92 circRNAs were upregulated and 44 were downregulated (Figure 4B), while at 2 days after adipocyte differentiation, 7 circRNAs were found to be differentially expressed, with 6 upregulated and 1 downregulated (Figure 4A). This indicated that more circRNAs were upregulated at day 12, compared to day 2 after adipocyte differentiation. 

### 3.4. Quantitative Real-Time PCR Verification

**Figure 5:**
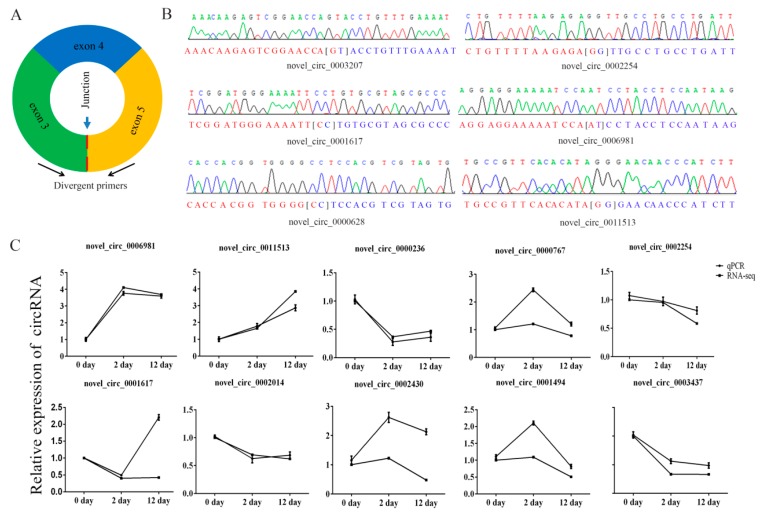
Verification of putative circRNAs. (**A**) Schematic illustration of the design of primers for circRNAs used in qRT-PCR. (**B**) Sanger sequencing confirmed back splicing site of representative circRNAs. (**C**) Change in circRNA levels between day 0, day 2, and day 12 groups. Day 2/day 0 and day 12/day 0 ratios for 10 circRNAs were based on the RNA-seq data and the expression of differentially expressed circRNAs as determined by qRT-PCR (three biological replicates, each done in triplicate).

We selected ten circRNAs and used their sequence-specific qPCR primers (Figure 5A) to detect differential expression of circRNAs and amplified the junction regions. The circRNAs junctions were verified by Sanger sequencing of the amplified PCR products (Figure 5B). The qPCR expression trends of circRNAs were similar to RNA sequencing data (Figure 5C), which indicated that the sequencing data were reliable.

### 3.5. GO and KEGG Pathway Analysis the Host Genes of circRNAs

**Figure 6:**
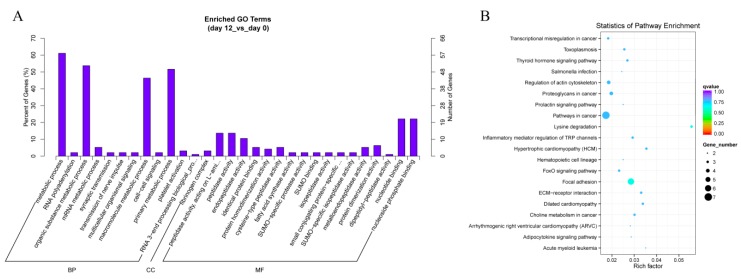
Annotations and enrichment of differentially expressed circRNAs. (**A**) GO analysis shows significantly (day 12 *vs.* day 0) enriched terms (*p* < 0.05) in biological process, molecular function, and cellular component categories. (**B**) KEGG pathway analysis shows differentially expressed (day 12 *vs.* day 0) circRNAs enriched in the adipocytokine signaling pathway, extracellular matrix (ECM)-receptor interactions, forkhead box O (FoxO) signaling pathway, focal adhesion, lysine degradation, inflammatory mediator regulation of transient receptor potential (TRP) channels, thyroid hormone signaling pathway, and regulation of the actin cytoskeleton.

In order to explore the biological functions of differentially expressed circRNAs, we performed GO analysis for the host genes of circRNAs. GO analysis classified host linear transcripts in biological process (BP), cellular components (CC) and molecular function (MF) after 12 days of yak adipocyte differentiation, as shown in Figure 6A. The prediction terms (*p*-value ≤ 0.05) for processes were screened and ranked according to their *p*-value. Our findings showed that genes involved in every GO category were connected with the RNA polyadenylation, metabolic process, peptidase activity, and nucleic binding, which suggested that several circRNAs played a main role in the basic process of adipocyte differentiation. As shown in Figure 6B, the KEGG pathways were enriched in the adipocytokine signaling pathway, forkhead box O (FoxO) signaling pathway, extracellular matrix (ECM)-receptor interactions, focal adhesion, lysine degradation, inflammatory mediator regulation of transient receptor potential (TRP) channels, thyroid hormone signaling pathway, and regulation of actin cytoskeleton, these were all concerned with adipocyte differentiation and proliferation, which revealed that circRNAs might play a pivotal role in adipocyte differentiation. After 2 days of yak adipocyte differentiation, GO and KEGG results were as shown in Supplementary Figure S1A–B, respectively. The most enriched GO terms were serine-type endopeptidase inhibitor activity, endopeptidase regulator activity, as well as endopeptidase inhibitor activity. The most enriched KEGG pathways were acute myeloid leukemia, transcriptional misregulation, and pathways in cancer.

### 3.6. Identification of CircRNA-MiRNA Axis/Pairs

The previous studies on functions of circRNAs were mainly focused on whether circRNAs could work as miRNA sponges to modulate the gene expression or not [10,23,24]. In this study, we performed miRanda to reveal that 131,884 interactions existed in the numerous miRNAs and 7203 circRNAs (Supplementary Table S4). Interestingly, we found that some well-known miRNAs were strongly associated with the differentiation of adipocytes. We considered that they would be promising candidates for subsequent research. CircRNAs (novel_circ_0009127, novel_circ_0000628, novel_circ_0011513, and novel_circ_0006981) had various potential target sites for adipocyte differentiation-related miRNAs (miR-143, miRNA-378, miRNA-328, and miR-34a, respectively). Subsequently, we further analyzed the interaction of miRNAs and circRNAs (Table 1). These results indicated that the process of adipocyte differentiation might be affected by the circRNAs.

### 3.7. Construction of the circRNA-miRNA-Gene Network

**Figure 7:**
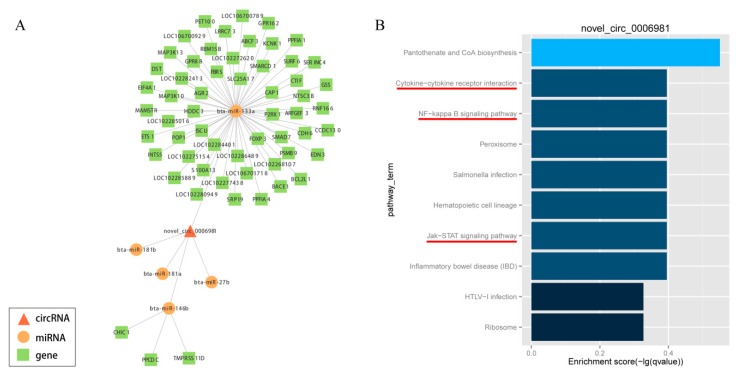
CircRNA-miRNA-gene network with potentially effective novel_circ_0006981 (**A**) and KEGG pathway analysis (**B**). Circle nodes represent miRNAs, rectangle nodes represent genes, and triangle nodes represent circRNAs.

In order to elucidate the competing endogenous RNA network, the targets of differentially expressed circRNAs and downstream-regulated genes were predicted by miRanda, which was formed on the basis of circRNA-miRNA-gene connectivity (Supplementary Table S5). Further, to explore the bio-function of circRNAs involved in adipocyte differentiation, we used Cytoscape software (version 3.5.1, BiNGO plug-in) to construct a competing endogenous network of circRNA-miRNA-gene, which was based on the underlying effect of circRNAs (novel_circ_0006981, novel_circ_0009127, novel_circ_0000628, novel_circ_0011513, novel_circ_0010775 and novel_circ_0001494), and their putative miRNA targets and downstream-regulated mRNAs (Figure 7A; Supplementary Figures S2–S6). The potentially significant circRNAs contain numerous binding sites for the miRNAs associated with adipogenic differentiation. Besides, the function of putative mRNA targets in the networks were annotated by KEGG pathway, as indicated in Figure 7B and Supplementary Figures S2–S6, which showed the KEGG pathways were enriched in phosphatidylinositol signaling system, nuclear factor kappa B (NF-kappa B) signaling pathway, Hedgehog signaling pathway, ECM-receptor interactions, steroid biosynthesis, pancreatic secretion, glycerolipid metabolism, cytokine-cytokine receptor interaction, Janus kinase (Jak)- signal transducer and activator of transcription (STAT) signaling pathway, Type II diabetes mellitus, FoxO signaling pathway and VEGF signaling pathway, these were all concerned with adipocyte differentiation and proliferation. This information provided a significant theoretical basis and reference for us to investigate the underlying mechanism of circRNA in the differentiation of yak adipocyte. Taken together, circRNAs may play an extensive endogenous regulatory role in the differentiation of yak adipocytes by interacting with multiple miRNAs. As a marked potential ceRNA, Novel circ 0006981 which controls adipocyte differentiation will be explored in further studies.

### 3.8. Homology Analysis of Yak circRNAs

**Figure 8:**
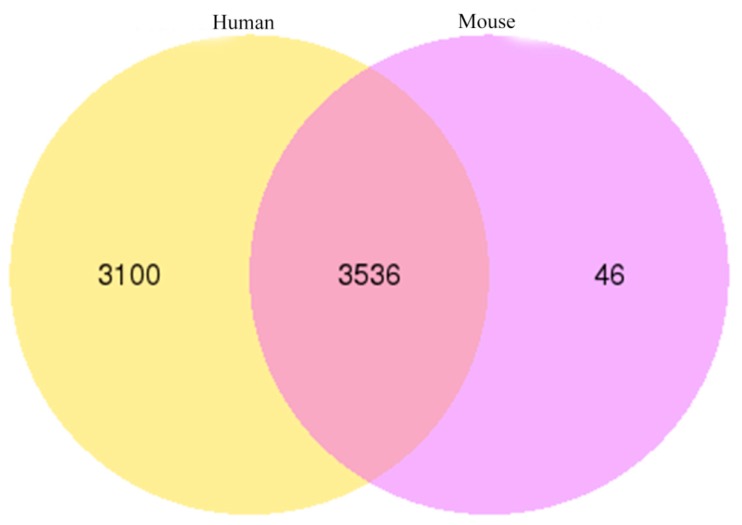
Conservation analysis of circRNAs among yak, human and mouse.

The circ-RNAs of yak, compared with human and mouse, showed that the yak circRNAs shared 6636 (92.13%) and 3582 (49.73%) homology with human and mouse circRNAs, respectively. Most of the yak circRNAs displayed high homology with these two species, especially with a human. In total, 3536 yak circRNAs showed homology with human and mouse circRNAs (Figure 8).

## 4. Discussion

The proliferation and differentiation of animal cells are mediated by a variety of regulatory factors, including growth factors, transcription factors, regulatory proteins and some hormone receptors, and so on. Previous studies on molecular mechanisms mainly focused on DNA, mRNA, and miRNA levels. Recently, the non-coding RNA of circRNA has changed its status from a rare curiosity to the central regulatory role in cell metabolism [10,24,25]. So far, transcriptome sequencing as a preferred biotechnique permits us to identify the circRNAs in diverse species [26,27,28,29,30], some of the circRNAs proved to play a vital role in animal growth and development. Recently,circRNA expression profiles have been characterized in adipose tissues of swine [31], mice [32], humans [33,34], buffalo [35] and cattle [28]. Nevertheless, the circRNAs in yak remain unknown. In order to fully understand the circRNAs related to bovine adipogenesis, in this study, we used RNA-seq to analyze the non-coding regions of primary cultured yak pre-adipocytes and adipocytes and successfully identified 7203 circRNAs among numerous circRNAs.

The continued discoveries of functional circRNAs showed that circRNAs as a transcriptional product had an essential role in various tissues and cell types of animals [8,31,36]. A total of 136 and 14 differentially expressed circRNAs were identified at day 2 and 12 after yak adipocyte differentiation and pre-adipocyte differentiation at day 0, respectively. These circRNAs might have potential biological function in yak adipocytes differentiation. Adipocytes proliferation and differentiation involve numerous differentially expressed genes and non-coding RNAs [37,38,39,40]. For instance, Li et al. [11] reported that lncRNA ADNCR suppressed cattle adipocyte differentiation by inhibiting miR-204 as a competitive endogenous RNA, and Chen et al. [41] elucidated that Lnc-U90926 attenuated 3T3-L1 adipocytes differentiation by suppressing the transactivation of PPARγ2 or PPARγ. Recently, Li et al. [31] reported that 275 circRNAs were differentially expressed in subcutaneous adipose tissues of Laiwu and Large White pigs and Sun et al. [42] identified 4080 circRNAs were significantly expressed in human visceral preadipocytes and adipocyte. Additionally, Huang et al. [35], identified a total of 5141 circRNAs in buffalo, among them 252 circRNAs were differentially expressed between the adult and young buffaloes and concluded that circRNAs strongly correlated with fat deposition associated genes. These findings suggested that non-coding RNAs possibly participated in the development of animal adipose tissue and adipocyte differentiation through post-transcriptional regulation. Thus, we hypothesized that circRNAs might play a key role in post-transcriptional regulation during yak adipocyte differentiation.

There is mounting evidence to indicate that circRNAs, through the mechanism of competitive endogenous RNAs (ceRNAs), modulate the function of miRNAs [10,24,43]. CircRNAs can absorb miRNAs, which through the way impact post-transcriptional regulation [44,45,46]. Wei et al. reported that circLMO7 regulates the differentiation and survival of myoblasts in cattle through sponging miR-378a-3p [28]. Likewise, Li et al. disclosed that CircFUT10 inhibits proliferation and promotes differentiation of cattle myoblast via sponging miR-133a [9]. Additionally, Liu et al. found that circular RNA SAMD4A controls adipogenesis through the miR-138-5p/EZH2 Axis in obesity [47]. Therefore, in the present study, we employed miRanda to explore the circRNAs target sites with miRNAs. Many circRNAs interacted with numerous miRNAs related to the development of adipose (e.g., miR-26a, miR-378, miR-146b and miR-138). Interestingly, it was found that several circRNAs contained target sites for many miRNAs. In our study, bta-miR-378 and bta-miR-200a were targets of novel_circ_0000628. Liu et al. reported that miR-378 promoted the differentiation of bovine pre-adipocytes by two targets E2F2 and RANBP10 [48]. Our previous study implicated that miR-200a regulated adipogenic in domestic yak adipocyte [49]. Likewise, novel_circ_0009127, novel_circ_0010775, novel_circ_0001494 and novel_circ_0011513 interacted with adipose related miRNAs (miR-130a, miR-130b, miR-143, miR-146b, miR-302a, miR-377 and miR-34a) [37,50,51,52,53,54]. In particular, our findings are consistent with those of [30], who reported that circHIPK3 regulated human cell growth via absorbing 9 miRNAs at 18 potential binding sites. Novel_circ_0006981 contained target sites of bta-let-7a-5p, bta-let-7e, bta-let-7f, bta-let-7g, bta-let-7i bta-miR-129-3p, bta-miR-133a, bta-miR-146b, bta-miR-181a, bta-miR-181b, bta-miR-181d, bta-miR-2459, bta-miR-27a-3p, bta-miR-27b, bta-miR-3600, bta-miR-361, bta-miR-432, bta-miR-98, and novel_620, simultaneously. Furthermore, another study presented that miR-181a promotes adipogenesis by targeting TNF-α in porcine adipocyte [55]. Dramatically, pathway analysis showed that the target genes of novel_circ_0006981 were enriched in NF-kappa B signaling pathway. Laurencikiene, J et al. [56] reported that NF-kappa B was important for TNF-a-induced lipolysis in human adipocytes. Hence, the circRNAs might play a comprehensive endogenous regulatory role through interaction with multiple miRNAs during the differentiation of yak adipocytes. Novel_circ_0006981, as a marked potential ceRNA, may regulate differentiation of adipocytes, which will be investigated in further studies. 

Previous findings revealed that several biological pathways participate in adipocyte differentiation, including the signaling pathway of AMP-activated protein kinase (AMPK), Wnt, Hedgehog, insulin-like growth factor (IGF), adipocytokine, FoxO, mammalian target of rapamycin (mTOR), as well as ECM-receptor interactions [57,58,59,60,61,62]. However, there is limited knowledge available related to the function of circRNAs in yak adipocytes and adipose tissue. Our research clearly shows that circRNAs played a vital role in regulating the yak adipocytes differentiation by KEGG enriched pathways. Mainly, the phosphatidylinositol signaling system, NF-kappa B signaling pathway, Hedgehog signaling pathway, ECM-receptor interactions, steroid biosynthesis, pancreatic secretion, glycerolipid metabolism, cytokine-cytokine receptor interaction, Jak-STAT signaling pathway, and VEGF signaling pathway were significantly enriched.

Homologous analysis of circRNAs is very important to reveal their common characteristics. The sequence of circRNAs shared high identity among different species [63,64]. In our research, BLAST was employed to analyze the homology of circRNAs. The results showed that 3582 and 6636 yak circRNAs were homologous to mouse and human, respectively. Our findings are in agreement with Guo et al., who reported that the mouse circRNAs were homologous to human circRNAs [65], and Sun et al. revealed that human hsa_circ_0094183 and hsa_circ_0116913 were homologous with mice MMU_CIRCpedia_216382 and MMU_CIRCpedia_14213 [42]. These results implied that circRNAs might have similar functions in eukaryotes.

## 5. Conclusions

In our paper, the comprehensive landscape of circRNA expression profile was identified during yak adipocyte differentiation. For the first time, a circRNA-miRNA-gene interaction network was established for plateau animals. Furthermore, several core networks were established as a base for miRNA target site in exons of circRNA loci analysis. Meanwhile, we explored several interesting circRNAs (novel_circ_0009127, novel_circ_0000628, novel_circ_0011513, novel_circ_0010775, novel_circ_0006981 and novel_circ_0001494), which functioned in NF-kappa B signaling pathway, Hedgehog signaling pathway, ECM-receptor interactions, steroid biosynthesis, glycerolipid metabolism, cytokine-cytokine receptor interaction, Jak-STAT signaling pathway, Type II diabetes mellitus, FoxO signaling pathway and VEGF signaling pathway, these all were closely linked to adipocyte differentiation and proliferation. Undoubtedly, our findings will facilitate the research of the regulatory mechanism for circRNA in bovine adipogenesis, which may offer further insights into the adipocyte differentiation process and valuable resources for understanding the circRNA biology in plateau animals.

## Figures and Tables

**Table 1:** Potential miRNA (adipocyte differentiation-related miRNAs) targets of circRNAs.

CircRNA ID	Regulation (day 12 *vs.* day 0)	Gene Symbol	Potential miRNA Targets
novel_circ_0000628	down	ADGRE5	bta-miR-140,bta-miR-148a,bta-miR-148b,bta-miR-152, bta-miR-191,bta-miR-200a,bta-miR-2284t-3p,bta-miR-2285aa, bta-miR-3120,bta-miR-378,bta-miR-378c,bta-miR-708, bta-miR-769
novel_circ_0009127	up	USP7	bta-miR-10a,bta-miR-10b,bta-miR-130a,bta-miR-130b, bta-miR-133a,bta-miR-143,bta-miR-148a,bta-miR-148b, bta-miR-152,bta-miR-22-5p,bta-miR-2285af,bta-miR-301a, bta-miR-3120,bta-miR-454,bta-miR-6518,bta-miR-95, novel_457
novel_circ_0010775	up	CEP85L	bta-miR-146b,bta-miR-15a,bta-miR-15b,bta-miR-16a, bta-miR-16b,bta-miR-1839,bta-miR-185,bta-miR-195, bta-miR-212,bta-miR-2400,bta-miR-2898,bta-miR-302a, bta-miR-31,bta-miR-3120,bta-miR-323,bta-miR-424-5p, bta-miR-497,bta-miR-677,bta-miR-7857,bta-miR-877, novel_367
novel_circ_0011513	up	HOMER1	bta-miR-193a-3p,bta-miR-22-5p,bta-miR-2411-5p, bta-miR-34a,bta-miR-3600,bta-miR-370,bta-miR-376a, bta-miR-376d,bta-miR-409b,bta-miR-449a,bta-miR-449b, bta-miR-449c,bta-miR-455-3p
novel_circ_0006981	up	IL1RL1	bta-let-7a-5p,bta-let-7e,bta-let-7f,bta-let-7g,bta-let-7i, bta-miR-129-3p,bta-miR-133a,bta-miR-146b,bta-miR-181a, bta-miR-181b,bta-miR-181d,bta-miR-2459,bta-miR-27a-3p, bta-miR-27b,bta-miR-3600,bta-miR-361,bta-miR-432, bta-miR-98,novel_620

## Supplementary Materials

The following are available online at https://www.mdpi.com/2073-4425/11/4/414/s1, Figure S1: Annotations and enrichment of differentially expressed circRNAs between day 2 and day 0, Figure S2: CircRNA-miRNA-gene network with potentially effective novel_circ_0009127 (A) and KEGG pathway analysis (B), Figure S3: CircRNA-miRNA-gene network with potentially effective novel_circ_0000628 (A) and KEGG pathway analysis (B), Figure S4: CircRNA-miRNA-gene network with potentially effective novel_circ_0011513 (A) and KEGG pathway analysis (B), Figure S5: CircRNA-miRNA-gene network with potentially effective novel_circ_0010775 (A) and KEGG pathway analysis (B), Figure S6: CircRNA-miRNA-gene network with potentially effective novel_circ_0001494 (A) and KEGG pathway analysis (B), Table S1: Sequence specific primers were used in this study. F: forward, R: reverse. β-actin was used as endogenous control genes for miRNA and mRNA., Table S2: CircRNA-seq quality control statistics., Table S3: Distribution of circRNAs in the genome based on the overlapping sequences of circRNAs on chromosomes, Table S4: Interaction relationships between circRNAs and various miRNAs were found, Table S5: The circRNA-miRNA-gene interaction network between circRNA and its target gene.
