# Supplementary material for: CircRNA Expression Profile during Yak Adipocyte Differentiation and Screen Potential circRNAs for Adipocyte Differentiation
Source: Genes (Basel). 2020 Apr 10;11(4):414. doi: 10.3390/genes11040414 (PMC7230347; doi:10.3390/genes11040414)
Supplement: Supplementary file 1 [file genes-11-00414-s001.zip › Supplementary files/Table S2.docx]

**Table s2.** CircRNA-seq quality control statistics.

| **Sample** | **Raw Reads** | **Clean Reads** | **Clean Bases** | **Error Rate (%)** | **Q20 (%)** | **Q30 (%)** | **GC Content (%)** |
| --- | --- | --- | --- | --- | --- | --- | --- |
| Ad_1 | 105520698 | 100919558 | 15.14G | 0.01 | 97.71 | 93.93 | 50.47 |
| Ad_2 | 109333974 | 105155332 | 15.77G | 0.01 | 97.58 | 93.68 | 49.81 |
| Ad_3 | 101697910 | 97354746 | 14.6G | 0.01 | 97.59 | 93.7 | 49.58 |
| Pread0_1 | 127116388 | 122564448 | 18.38G | 0.01 | 97.86 | 94.31 | 49.67 |
| Pread0_2 | 101634046 | 97689334 | 14.65G | 0.01 | 97.63 | 93.79 | 54.83 |
| Pread0_3 | 101150218 | 96181158 | 14.43G | 0.01 | 97.72 | 93.97 | 55.96 |
| Pread2_1 | 125445142 | 120859972 | 18.13G | 0.01 | 97.74 | 94.03 | 51.71 |
| Pread2_2 | 117619462 | 113379914 | 17.01G | 0.01 | 97.79 | 94.13 | 51.18 |
| Pread2_3 | 110949568 | 106910494 | 16.04G | 0.01 | 97.54 | 93.59 | 50.49 |
