# Supplementary figures and images for: CircRNA Expression Profile during Yak Adipocyte Differentiation and Screen Potential circRNAs for Adipocyte Differentiation
Source: Genes (Basel). 2020 Apr 10;11(4):414. doi: 10.3390/genes11040414 (PMC7230347; doi:10.3390/genes11040414)

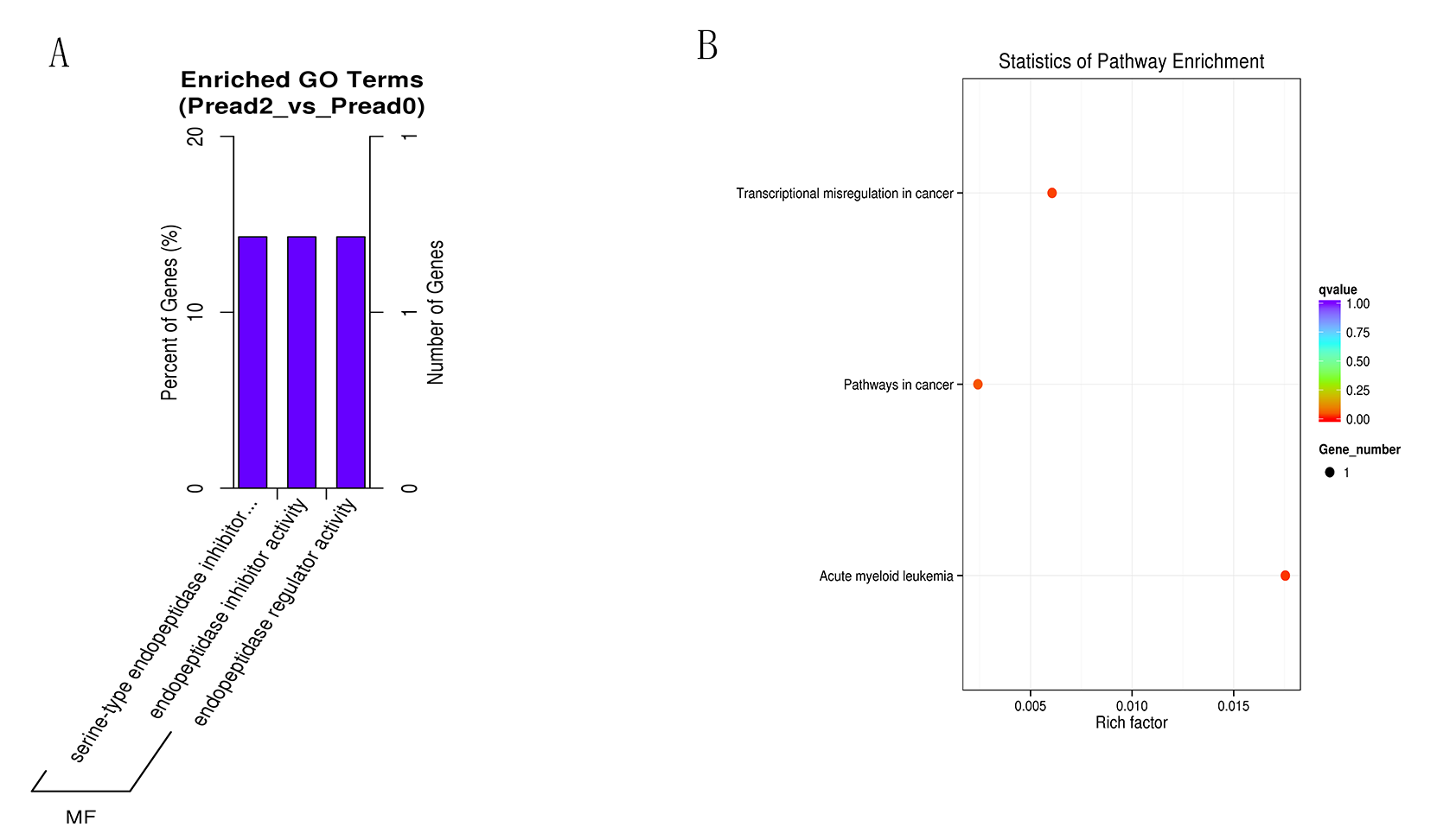

Supplement: Supplementary file 1 [file genes-11-00414-s001.zip › Supplementary files/Figure S1.tif]

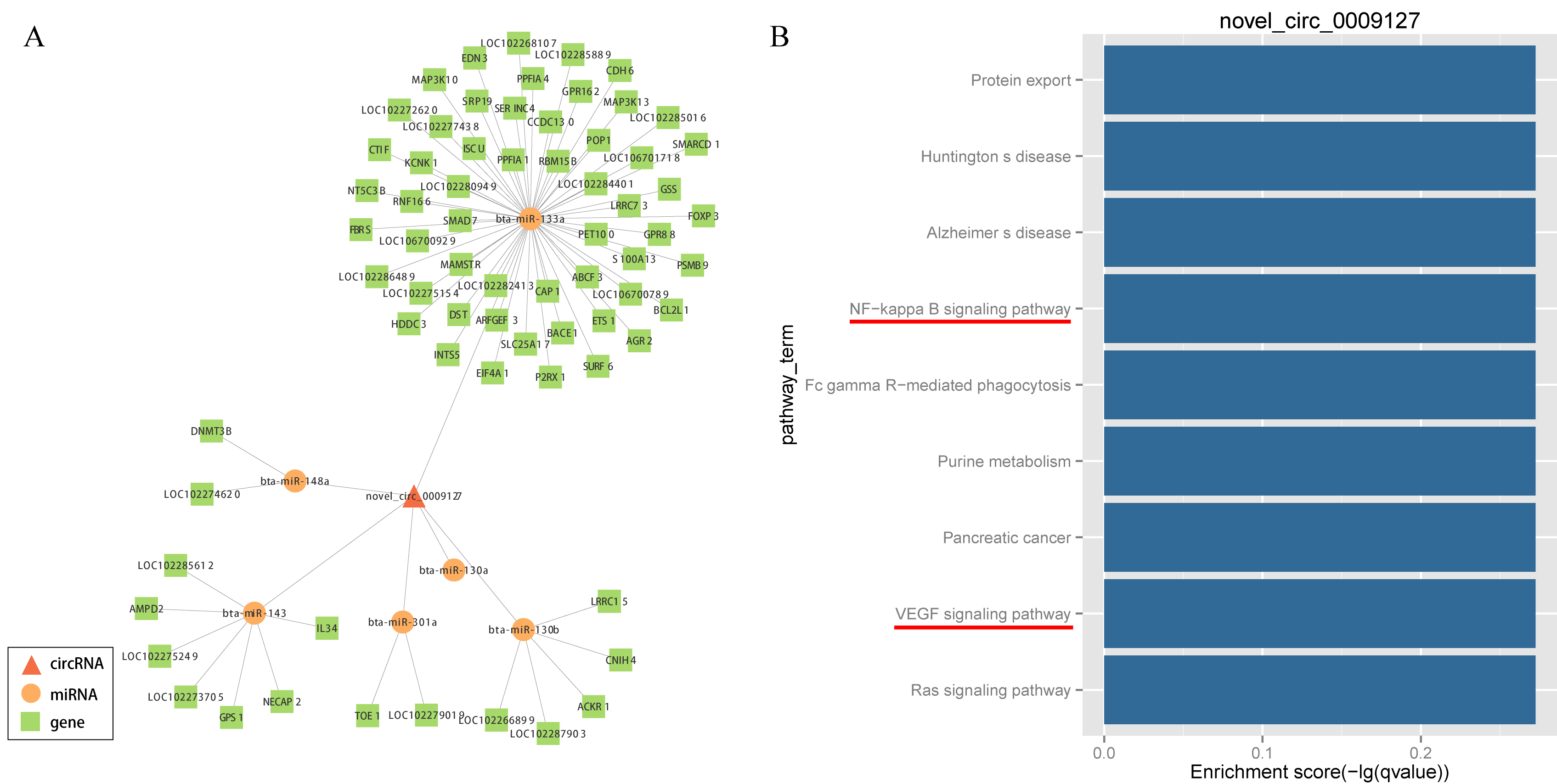

Supplement: Supplementary file 1 [file genes-11-00414-s001.zip › Supplementary files/Figure S2.tif]

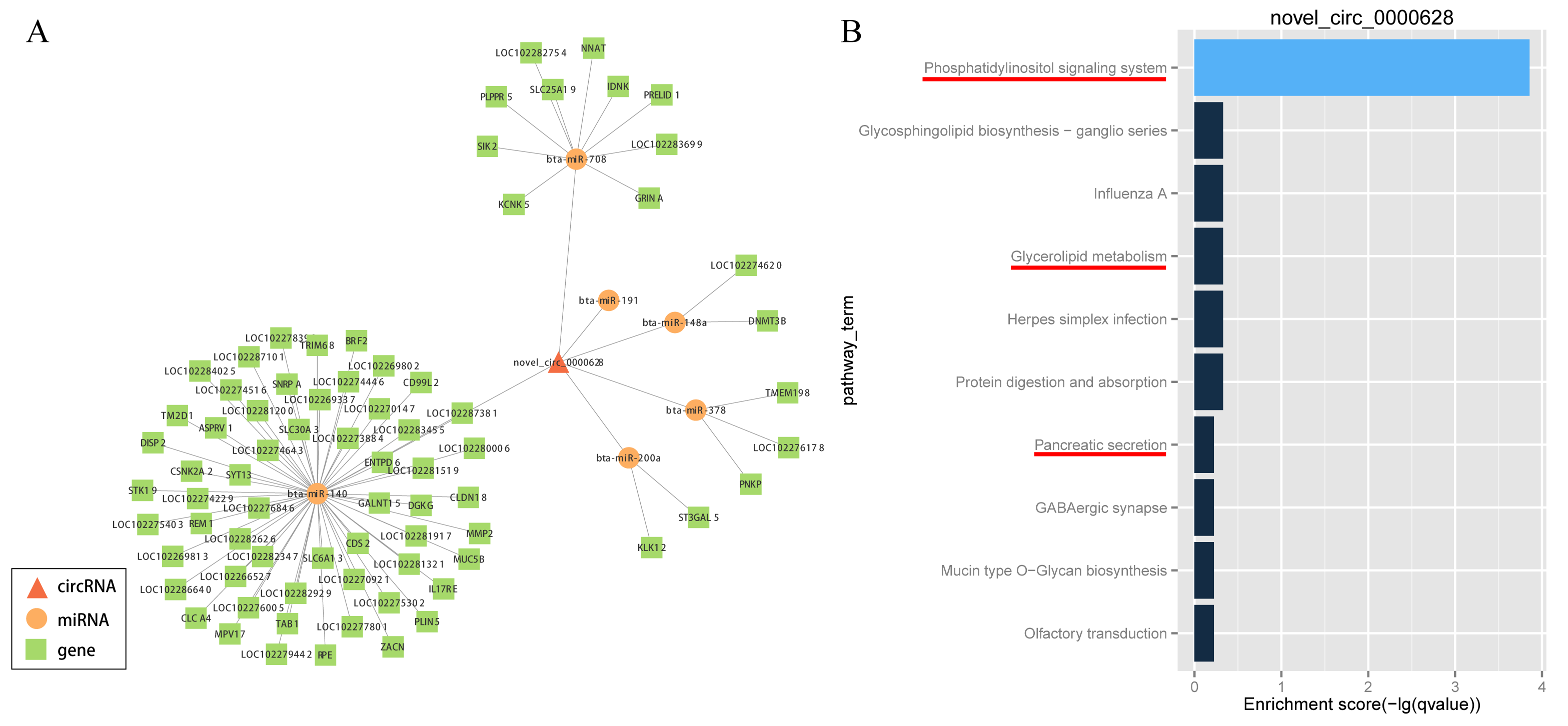

Supplement: Supplementary file 1 [file genes-11-00414-s001.zip › Supplementary files/Figure S3.tif]

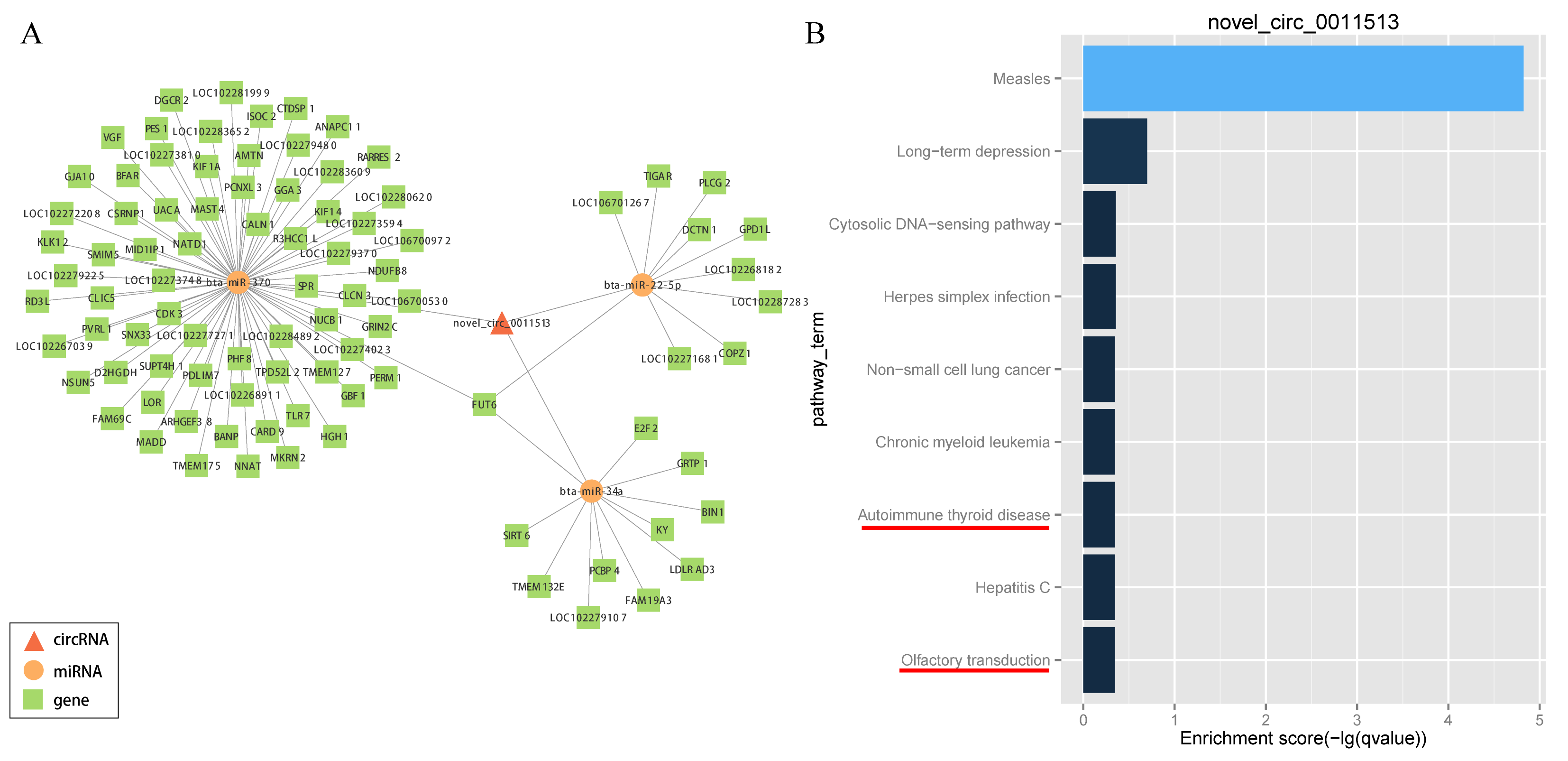

Supplement: Supplementary file 1 [file genes-11-00414-s001.zip › Supplementary files/Figure S4.tif]

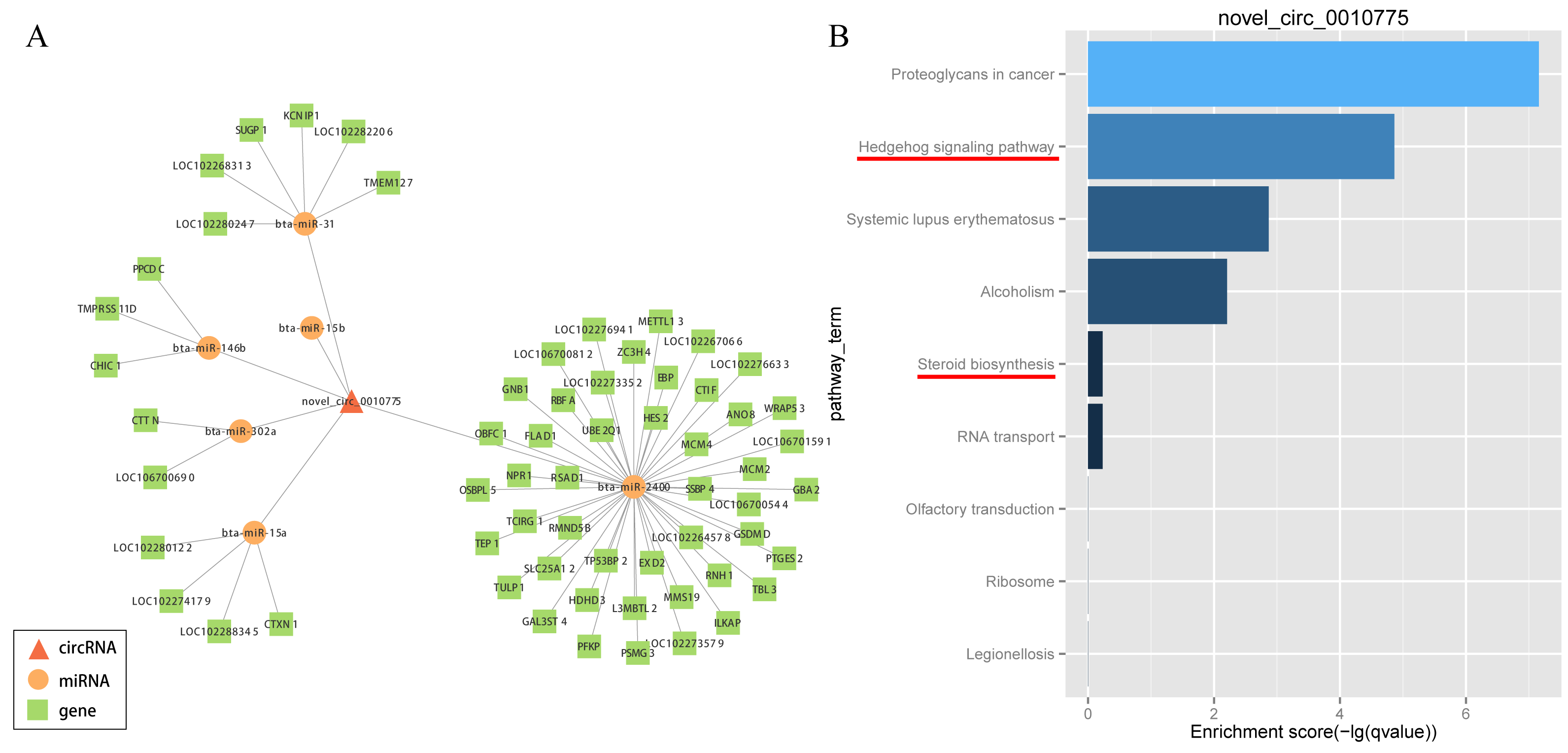

Supplement: Supplementary file 1 [file genes-11-00414-s001.zip › Supplementary files/Figure S5.tif]

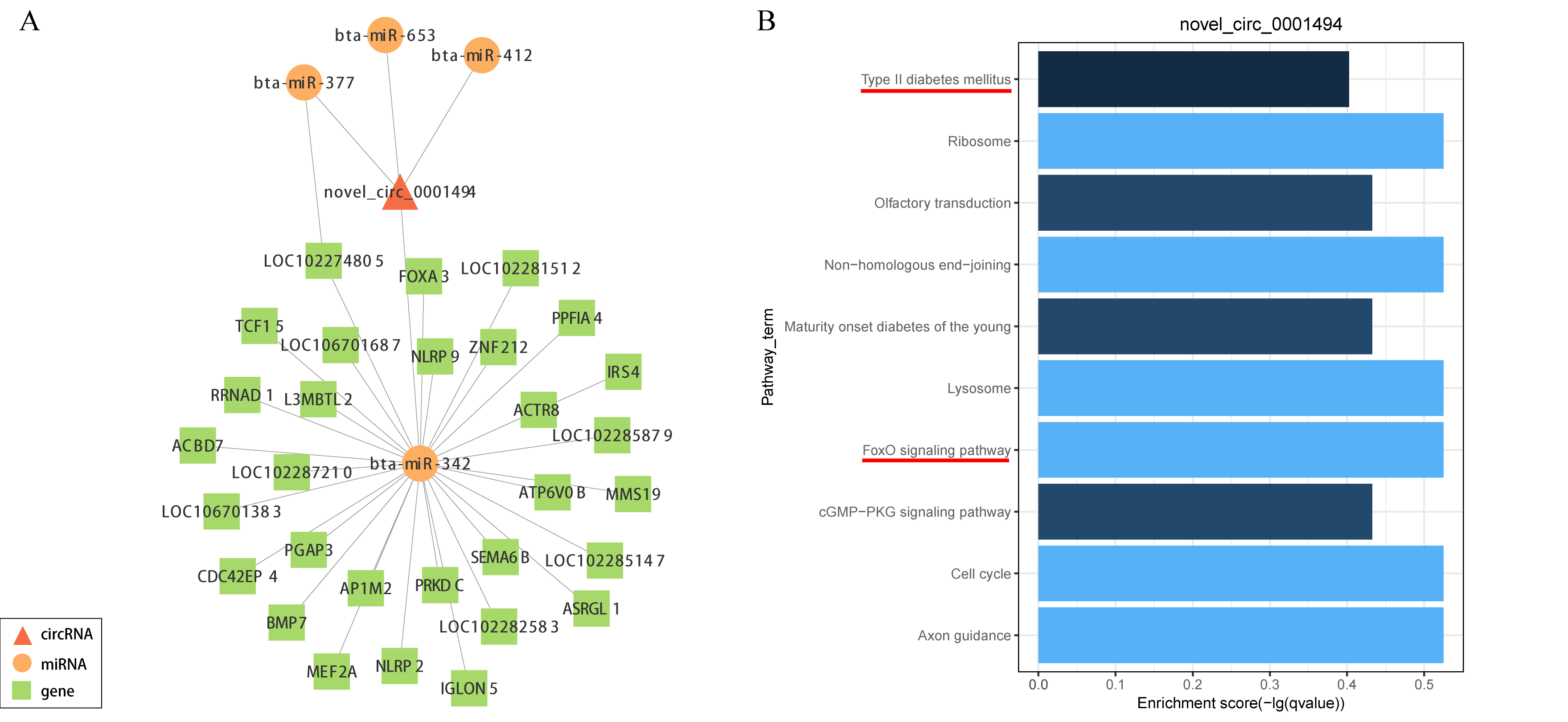

Supplement: Supplementary file 1 [file genes-11-00414-s001.zip › Supplementary files/Figure S6.tif]
